# Strong isolation by distance among local populations of an endangered butterfly species (*Euphydryas aurinia*)

**DOI:** 10.1002/ece3.8027

**Published:** 2021-08-13

**Authors:** Cino Pertoldi, Aritz Ruiz‐Gonzalez, Simon Bahrndorff, Nanna Renee Lauridsen, Thøger Nisbeth Henriksen, Anne Eskildsen, Toke Thomas Høye

**Affiliations:** ^1^ Department of Chemistry and Bioscience Aalborg University Aalborg Denmark; ^2^ Aalborg Zoo Aalborg Denmark; ^3^ Department of Zoology and Animal Cell Biology University of the Basque Country UPV/EHU Vitoria‐Gasteiz Spain; ^4^ Department of Bioscience and Arctic Research Centre Aarhus University Rønde Denmark

**Keywords:** fragmentation, genetic drift, genotyping‐by‐sequencing, inbreeding, SNP

## Abstract

The marsh fritillary (*Euphydryas aurinia*) is a critically endangered butterfly species in Denmark known to be particularly vulnerable to habitat fragmentation due to its poor dispersal capacity. We identified and genotyped 318 novel SNP loci across 273 individuals obtained from 10 small and fragmented populations in Denmark using a genotyping‐by‐sequencing (GBS) approach to investigate its population genetic structure. Our results showed clear genetic substructuring and highly significant population differentiation based on genetic divergence (*F*_ST_) among the 10 populations. The populations clustered in three overall clusters, and due to further substructuring among these, it was possible to clearly distinguish six clusters in total. We found highly significant deviations from Hardy–Weinberg equilibrium due to heterozygote deficiency within every population investigated, which indicates substructuring and/or inbreeding (due to mating among closely related individuals). The stringent filtering procedure that we have applied to our genotype quality could have overestimated the heterozygote deficiency and the degree of substructuring of our clusters but is allowing relative comparisons of the genetic parameters among clusters. Genetic divergence increased significantly with geographic distance, suggesting limited gene flow at spatial scales comparable to the dispersal distance of individual butterflies and strong isolation by distance. Altogether, our results clearly indicate that the marsh fritillary populations are genetically isolated. Further, our results highlight that the relevant spatial scale for conservation of rare, low mobile species may be smaller than previously anticipated.

## Graphical abstract

The marsh fritillary (Euphydryas aurinia) is a critically endangered butterfly species in Denmark known to be particularly vulnerable to habitat fragmentation due to its poor dispersal capacity. We identified and genotyped 318 novel SNP loci across 273 individuals obtained from 10 populations in Denmark using a genotyping‐by‐sequencing (GBS) approach to investigate its population genetic structure. Altogether, our results clearly indicate genetic substructuring and reduced gene‐flow among populations of the marsh fritillary, which are seriously affecting the genetic variability and level of inbreeding present.

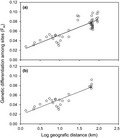

## 1 INTRODUCTION

Habitat loss and fragmentation represent a fundamental challenge for the conservation of biodiversity (Fahrig, 2003; Haddad et al., 2015). Increased isolation of habitat patches can increase dispersal‐related mortality and affect gene flow (Young et al., 1996). Although the genetic effects of habitat fragmentation are not straightforward, increased genetic differentiation is normally expected in species with poor dispersal capacity (e.g., Barluenga et al., 2011; but see Gu et al., 2015). As a result, populations separated by unsuitable habitat where only occasional dispersal events from one patch to another occur may exhibit a pattern of isolation by distance. Very few case studies have examined whether isolation by distance is maintained at spatial scales comparable to the dispersal capacity of individuals (Manel & Holderegger, 2013). In such cases, the number and the quality of the genetic markers used to detect isolation by distance are obviously of critical importance (Kool et al., 2013). Dramatic increases in computing power and exponential expansion in the availability of genetic markers have shed light on new opportunity for identifying patterns of genetic connectivity (Lowe & Allendorf, 2010). The choice of the genetic marker is crucial for the determination of the connectivity patterns over long time periods. There are, however, several challenges, as variability is introduced into the data (due to life‐history characteristics and population processes like mortality and birth rate). The mutation rate of the genetic marker is also clearly playing a role and determines the level of resolution (Ouborg et al., 2010).

Conserving genetic diversity is a key priority for biodiversity management to ensure adaptive potential, yet population fluctuations lead to low effective genetic population size (Joyce & Pullin, 2003). Hence, rare species typically have reduced genetic diversity due to low effective population size (Fraser et al., 2014), but see Wood et al. (2015). At the same time, habitat fragmentation prevents species with low mobility from building large population sizes. Therefore, rare species with low mobility are particularly likely to exhibit strong genetic differentiation and isolation by distance (Putz et al., 2015).

By quantifying genetic differentiation among populations, it is possible to assess the spatial dynamics of low‐mobility species, and with sufficient resolution, it is even possible to identify spatial clusters of populations suitable for conservation actions. Conservation planning for rare species will benefit substantially from knowledge of the relationship between genetic differentiation and geographic distance. A correlation between geographic distance and genetic divergence has often been used as evidence for isolation by distance (Slatkin, 1993). The lack of a correlation implies that the barriers among populations are considerably reducing or interrupting gene flow among the populations and that consequently the degree of divergence among populations is governed by genetic drift. With such information, spatial conservation units can be identified and informed decisions can be made about where to prioritize connectivity among habitats and where to expand a species range by forming new habitat networks (Kukkala & Moilanen, 2013; Lehtomaki & Moilanen, 2013).

The marsh fritillary (*Euphydryas aurinia*) butterfly species is likely to be a case in point. This species has a short flight season in May–June and females emerge with several hundred fully developed eggs, mate shortly after emergence, and typically lay these eggs within meters of their pupation site. Under good conditions, females can produce additional egg batches that may be laid further from the emergence site, but dispersal in the adult stage is mostly rather limited. Similarly, the larvae, which overwinter in family clusters, only move short distances and mostly in the final instar in spring prior to pupation (Porter & Ellis, 2011). This species is listed in the Annex II of the EU Habitats Directive and has experienced considerable range contraction in Europe (Warren, 1994) as well as in Denmark over the past 100 years (Eskildsen et al., 2015). It was once a widespread species in Denmark, but has since declined dramatically and was considered virtually extinct in Denmark around year 2000 (Asbirk & Christensen, 2000). Since then, it has been found again in a number of small patches in Northern Jutland partly as a result of conservation actions and increased efforts to search for remnant populations (Brunbjerg et al., 2017). A key question is, therefore, whether genetic diversity is maintained in a rare species like the marsh fritillary in a fragmented habitat like its range in Denmark.

**FIGURE 1:**
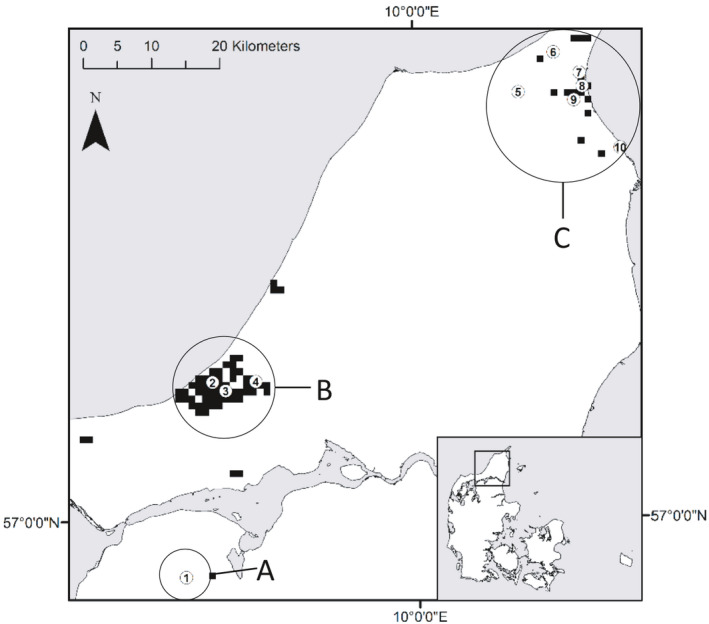
The current distribution of the marsh fritillary (*Euphydryas aurinia*) in Denmark as indicated by black 1 × 1 km grid cells on the main map of Northern Jutland where the species has been observed during (2004–2015). The region in which the marsh fritillary is found in Denmark is indicated on the inset map of the country. The populations sampled for this study cluster in the three main regions A, B, and C of the distribution of the species in Denmark. A few patches occur outside these main regions, but only with small population sizes at one or two sites. The exact location of each sampled population is indicated by a number referring to the following population names: (1) Bruså, (2) Tranum Skydeterræn, (3) Overklitten Sø, (4) Sandmosen, (5) Vågholt Mose, (6) Troldkærvej, (7) Knasborgvej, (8) Videsletengen, (9) Milrimvej, and (10) Strandby

The main threats to the marsh fritillary butterfly in Denmark are habitat loss, encroachment as a result of eutrophication and the cessation of management, drainage, and the indirect effects of artificial fertilizers and pesticides applied to agricultural fields in the surroundings of marsh fritillary habitat (heaths, marshes, and grasslands) (Tjørnløv et al., 2015). Several conservation programs have been carried out (a) to manage marsh fritillary habitat, (b) to monitor the species demography, and (c) to increase the public awareness of the species and its threats (Larsen, 2008). In 2000, the Danish Nature Agency developed the first management plan of the marsh fritillary butterfly, where the main objectives for the conservation of the species were listed (Asbirk & Christensen, 2000). Unfortunately, too few resources have been devoted to the implementation of the management plan. The distribution of the marsh fritillary in Denmark is fragmented and split into three main regions and a few additional small and isolated populations (Figure 1). Interpatch movement distances above three kilometers have previously been shown to be very rare for this species (Johansson et al., 2019). Dispersal among these three regions must, therefore, be very rare if it happens at all, and the potential for expansion into new areas is limited given the current habitat availability. According to the management plan, the maintenance of metapopulations of marsh fritillary should be the main priority, with the objective of maintaining a population size of at least 500 larval clusters per metapopulation. Recent demographic studies show that population sizes often fall far below this minimum (Lauridsen, 2015). The species can exhibit large fluctuations in population size due to weather conditions or parasites, which makes it very vulnerable to local extinctions (Joyce & Pullin, 2003). These fluctuations are also strongly affecting the effective population size which will tend to be approximately equivalent to the harmonic mean of the population size across years (Caballero, 1994).

A well‐established technique for the detection of the genetic structure is the genotyping‐by‐sequencing (GBS), which is a reproducible, highly multiplexed next‐generation sequencing approach that uses restriction enzymes to reduce genome complexity allowing for simultaneous single nucleotide polymorphism (SNP) discovery and genotyping (Elshire et al., 2011). The major advantages over other available protocols are both technical simplicity (Davey et al., 2011) and that informatics pipelines are publicly available and can be easily adapted to a wide variety of species, either with or without reference genome information (Elshire et al., 2011; Glaubitz et al., 2014). GBS, however, has not previously been used for SNP genotyping in any species of the Nymphalidae family. In the current study, we optimized the GBS protocol for the critically endangered marsh fritillary and genotyped a compressive number of individuals from 10 Danish populations. The resulting SNP dataset was used to analyze the genetic structure of the marsh fritillary in order to examine the spatial scale at which genetic differentiation can be detected for a rare, low mobile species.

We aim to answer the following questions (1) Is there evidence of isolation by distance in Danish populations of the marsh fritillary and at what spatial scales can it be detected? (2) Do populations with lower levels of inbreeding also show reduced genetic diversity? and (3) Are the levels of genetic variability and inbreeding related to the genetic isolation among populations?

## 2 MATERIALS AND METHODS

### 2.1 Sample collection

A total of 300 3rd or 4th instar larvae of the marsh fritillary were collected from 10 sites in Denmark (Figure 1) between August 14 and 31, 2014. At each site, one larva was collected from each of 30 larval clusters. Larvae were kept in separate containers and frozen immediately after collection in the field. The sampling sites were all less than one hectare of mostly homogenous habitat. The geographical coordinates of individual larval clusters within sites were not considered for this study. The locations were numbered as (1) Bruså, (2) Tranum Skydeterræn, (3) Overklitten Sø, (4) Sandmosen, (5) Vågholt Mose, (6) Troldkærvej, (7) Knasborgvej, (8) Videsletengen, (9) Milrimvej, and (10) Strandby, and the latitude and longitude of each site are given in Table 1. The marsh fritillary exists in three main geographical regions in Denmark, where population (1) belongs to region A, populations (2), (3), and (4) belong to region B and the remaining populations belong to region C (Figure 1, Table 1).

**TABLE 1:** Summary of population genetic data from each of the 10 Danish populations of the marsh fritillary (*Euphydryas aurinia*) investigated

Region	Population Name	ID	Latitude	Longitude	Parameter	N	P%	Ne	H_O_	H_E_	uH_E_	*F*_IS_
Region A	Bruså	Pop1	56°55.519′N	9°26.312′E	Mean	30	64.47	1.343	0.183	0.203	0.207	0.100
					SE			0.021	0.011	0.011	0.011	0.019
Region B	Tranum Skydeterræn	Pop2	57°10.948′N	9°30.338′E	Mean	30	81.13	1.368	0.194	0.228	0.232	0.141
					SE			0.018	0.009	0.010	0.010	0.018
	Overklitten Sø	Pop3	57°11.000′N	9°36.604′E	Mean	28	76.10	1.372	0.190	0.224	0.228	0.130
					SE			0.020	0.010	0.010	0.011	0.018
	Sandmosen	Pop4	57°33.647′N	10°15.496′E	Mean	28	72.33	1.365	0.188	0.221	0.225	0.126
					SE			0.020	0.010	0.010	0.011	0.017
Region C	Vågholt Mose	Pop5	57°34.596′N	10°24.516′E	Mean	25	85.22	1.376	0.189	0.237	0.242	0.179
					SE			0.017	0.009	0.009	0.009	0.019
	Troldkærvej	Pop6	57°33.899′N	10°24.938′E	Mean	17	74.21	1.351	0.179	0.216	0.224	0.137
					SE			0.019	0.010	0.010	0.010	0.021
	Knasborgvej	Pop7	57°29.112′N	10°30.345′E	Mean	30	85.85	1.367	0.190	0.231	0.235	0.142
					SE			0.018	0.009	0.009	0.009	0.017
	Videsletengen	Pop8	56°55.519′N	9°26.312′E	Mean	30	90.25	1.380	0.188	0.241	0.246	0.185
					SE			0.017	0.008	0.009	0.009	0.018
	Milrimvej	Pop9	57°10.275′N	9°32.226′	Mean	27	88.99	1.364	0.171	0.232	0.237	0.225
					SE			0.017	0.008	0.009	0.009	0.019
	Strandby	Pop10	57°11.000′N	9°36.604′E	Mean	28	84.28	1.362	0.170	0.225	0.229	0.208
					SE			0.018	0.008	0.010	0.010	0.018

Note

The region, population name, id, latitude, and longitude are given for reference. The number of individuals sampled in each population (*n*) and proportion of polymorphic loci (P%) are presented along with mean and standard error (SE) of mean effective alleles (Ne) based on 318 SNPs, observed heterozygosity (H_O_), expected heterozygosity (H_E_), unbiased expected heterozygosity (uH_E_), and inbreeding coefficient (*F*_IS_).

All the genomic analysis and the bioinformatic filtering of the data have been outsourced to the Cornell University Genomic Diversity Facility (US) (https://www.biotech.cornell.edu/core‐facilities‐brc/facilities/genomics‐facility).

### 2.2 DNA extraction and genotyping‐by sequencing (GBS) protocol optimization

The total genomic DNA was extracted from larvae using the DNeasy® Blood & Tissue Kit (Qiagen, Inc., Hilden, Germany) according to the manufacturer's protocol for purification of total DNA from insects. DNA quantity and quality was verified using a fluorometer (Qubit^®^, Thermo Fisher Scientific Inc.) and by running 100 ng of each DNA sample on a 1% agarose gel, respectively.

For optimization of the standard GBS protocol (Elshire et al., 2011) for *E. aurinia*, a single DNA sample (400 ng) was digested for 2 hr with the restriction enzymes ApeKI, EcoT22I, and PstI, in separate essays, using a tenfold excess of enzyme and reaction conditions as specified by the endonuclease manufacturer (New England Biolabs). After ligation of appropriate adapters (adapter amounts were determined by titration as described in REF(Elshire et al., 2011) and PCR (see below)), fragment size distributions of each test library were visualized using an Experion (Bio‐Rad, Hercules, California, USA) (Figure S1). Based on these results, we selected the libraries obtained from the *Eco*T22I digests to maximize sequence coverage from GBS.

### 2.3 Preparation of Illumina libraries for next‐generation sequencing

Three 96‐plex *Eco*T22I GBS libraries, comprising 285 DNA samples and three negative (no DNA) control, were prepared according to Elshire et al. (2011). Low DNA concentration samples (*n* = 15) were discarded and not submitted for sequencing. Briefly, individual DNA samples were digested with the restriction enzyme and adapters were ligated as described previously. The adapters comprised a set of 96 different barcodes containing adapters and a “common” adapter. Individual ligations were pooled, and purified using QIAquick PCR purification kit (Qiagen). Genomic fragments were then amplified in a 50‐μl volume containing 2‐μl pooled DNA fragments, 1× Taq Master Mix (New England Biolabs), and 25 pmol, each, of the following primers: (a) 5′‐AATGATACGGCGACCACCGAGATCTACACTCTTTCCCTACACGACGCTCTTCCGATCT‐3′ and (b) 5′‐CAAGCAGAAGACGGCATACGAGATCGGTCTCGGCATTCCTGCTGAACCGCTCTTCCGATCT‐3′. PCR cycling consisted of 72°C for 5 min, 98°C for 30 s, followed by 18 cycles of 98°C for 30 s, 65°C for 30 s, and 72°C for 30 s, with a final extension step at 72°C for 5 min. The *Eco*T22I GBS library was purified again, as above, and an aliquot was run on an Agilent BioAnalyzer 2100 for evaluation of fragment sizes and the presence of adapter dimers. After quantification on a Nanodrop 2000 (Thermo Scientific), the three 96‐plex libraries were diluted and sequenced (single‐end reads only; read length 1 × 100‐bp reads) using three lanes on the Illumina HiSeq 2000. GBS library preparation (Elshire et al., 2011), sequencing, and SNP calling were performed at the Genomic Diversity Facility (GDF) at Cornell University's Biotechnology Resource Center.

### 2.4 DNA sequence analysis: SNP discovery and genotyping

Illumina raw reads were processed using the default parameter of the Universal Network‐Enabled Analysis Kit (UNEAK) (Lu et al., 2013) for species without a reference genome. This pipeline was implemented in TASSEL version 3.0.166 (Glaubitz et al., 2014) and used for tag alignment and subsequent SNP calling. Briefly, the raw Illumina DNA sequence data (100‐bp qseq files) were first trimmed to remove barcodes. The sequence remnants were then either trimmed further or padded with 3’ A's to 64‐bp lengths. Sequences were then aligned to each other, both to identify unique sequences, or “sequence tags”, and to generate clusters of related sequences. For each cluster, a network was generated, in which sequence tags were organized according to mutation steps (i.e., mutational relationship). A single base‐pair mismatch was allowed among cluster members. Networks were then filtered such that only SNPs originating from reciprocal tag pairs were retained (see Lu et al., 2013). SNPs from more complicated networks that often result from alignment of paralogs and repeats, or sequencing errors were discarded. To further reduce the impact of sequencing errors, we also set the error tolerance rate (ETR) parameter to 0.03, slightly below the expected Illumina sequencing error rate (0.04%). Failed samples (nonblank), defined as those with less than 10% of the mean reads per sample coming from the lane on which they were sequenced, were discarded (*n* = 5).

The resulting raw SNP dataset from the UNEAK pipeline was further filtered using Golden Helix SNP and Variation Suite (SVS version 7.2.2, Golden Helix, Bozeman, MT) and PLINK v1.07 (Purcell et al., 2007) softwares. First, the dataset was filtered by the application of genotype‐level filters to remove genotypes with low read depths (RD) and/or low genotype quality (GQ). Thus, genotypes with RD ≤ 4× and GQ ≤ 98 were considered as missing. Later, we removed all SNPs and individuals with call rates <80%. In addition, SNPs with a minor allele frequency (MAF) < 0.05 were removed. Loci with a mean‐observed heterozygosity >0.6 were also discarded to filter out potential paralogs. The SNP set was also pruned for linkage disequilibrium (LD) by excluding markers in strong LD (pairwise genotype correlation *r* ^2^ > .5) in a window of 50 SNPs (sliding window overlap 10 SNPs at a time). This filtering process is described in Figure S2.

### 2.5 Genetic variability and population structure

Genetic variability in each population was assessed by the calculation of observed heterozygosity (H_O_), expected heterozygosity (H_E_), unbiased heterozygosity (uH_E_) and inbreeding coefficient (*F*_IS_), mean percentage polymorphic loci (%P), and mean effective alleles (Ne) were likewise calculated using GenAlEx 6.501 (Peakall & Smouse, 2012). Deviations from Hardy–Weinberg Equilibrium (HWE) (probability test) were analyzed using GENEPOP v4.3 on all the populations pooled together and on each population considered singularly (Rousset, 2008).

Population pairwise *F*_ST_ were calculated to reveal the genetic differentiation using GenAlEx 6.501, and Fisher's exact probability test for testing for genic differentiation was carried out using GENEPOP v4.3. For every population, the mean of pairwise *F*_ST_ values between the population and all other populations was considered as an index of isolation.

A maximum likelihood‐based clustering algorithm implemented in ADMIXTURE v1.23 (Alexander et al., 2009) was applied to the entire dataset to identify the putative ancestral cluster(s) within the samples as well as to assess the extent of genetic admixture. Clustering was performed 100 times for all K‐values from K = 2 to K = 12, and the best‐fitting K was selected based on the lowest cross‐validation error (CVE). Additionally, a principal component analysis (PCA) was performed in R (R Development Core Team https://www.r‐project.org/), based on the genetic distances using GenAlEx v6.501 to examine how populations would cluster along principal component axes 1 and 2. Finally, Mantel's tests were performed on all the populations investigated in order to determine whether there was an overall correlation between geographic distance and genetic divergence (Smouse et al., 1986). Some authors argue that spatial structures in the dataset can enhance isolation by distance (Legendre et al., 2015). To control for such a potential effect, we ran one test based on all pairwise comparisons and one test on a subset of pairwise comparisons excluding pairs from two different regions.

## 3 RESULTS

The UNEAK pipeline recovered 30,137 bi‐allelic SNP loci (*n* = 280; 5 samples failed the UNEAK pipeline). However, most of these SNPs had low coverage or were only present in a small number of individuals. After the complete filtering procedure, 318 SNPs were maintained in our matrix for 273 individuals with an overall call rate of 93.57% (see Figure S2). Over all of these loci, the mean coverage per locus per individual was 87.88 (max coverage per individual 207.21, min 8.21).

Deviations from HWE were found to be highly significant for all the 10 populations investigated (*p* < .001 in all cases), both when pooled altogether and when considered singularly. All the deviations were due to heterozygote deficiency as can be seen by the positive *F*_IS_ values ranging from 0.1 to 0.228 (Table 1). Genetic variability parameters, observed heterozygosity (H_O_), unbiased heterozygosity (uH_E_) and inbreeding coefficient (*F*_IS_), mean percentage polymorphic loci (%P), and mean effective alleles (Ne), are listed in Table 1. Genetic divergence between populations ranged from 0.028 to 0.1 (Table 2). All the pairwise *F*_ST_ values were highly significant (*p* < .001).

**TABLE 2:** Pairwise *F*_ST_ values (lower left) and geographical distance in kilometers (upper right) among all pairs of the 10 Danish populations of the marsh fritillary (*Euphydryas aurinia*) were investigated

	Pop1	Pop2	Pop3	Pop4	Pop5	Pop6	Pop7	Pop8	Pop9	Pop10
Pop1		28.9	28.0	30.5	86.3	94.1	93.2	92.5	90.3	89.7
Pop2	0.083		2.3	6.3	61.8	69.6	69.8	69.3	67.3	69.0
Pop3	0.092	0.036		4.6	61.3	69.1	69.2	68.7	66.6	68.0
Pop4	0.091	0.049	0.048		57.3	65.1	65.0	64.4	62.3	63.5
Pop5	0.086	0.065	0.074	0.078		7.8	9.2	9.4	8.2	17.0
Pop6	0.100	0.084	0.098	0.092	0.052		5.5	6.7	7.6	17.1
Pop7	0.088	0.067	0.074	0.083	0.034	0.053		1.4	3.2	11.7
Pop8	0.079	0.063	0.071	0.074	0.03	0.044	0.028		2.2	10.4
Pop9	0.082	0.066	0.072	0.077	0.037	0.051	0.041	0.031		9.8
Pop10	0.096	0.069	0.078	0.079	0.048	0.062	0.048	0.040	0.050	

Note

All *F*_ST_ comparisons were highly significant (*p* < .001).

**FIGURE 2:**
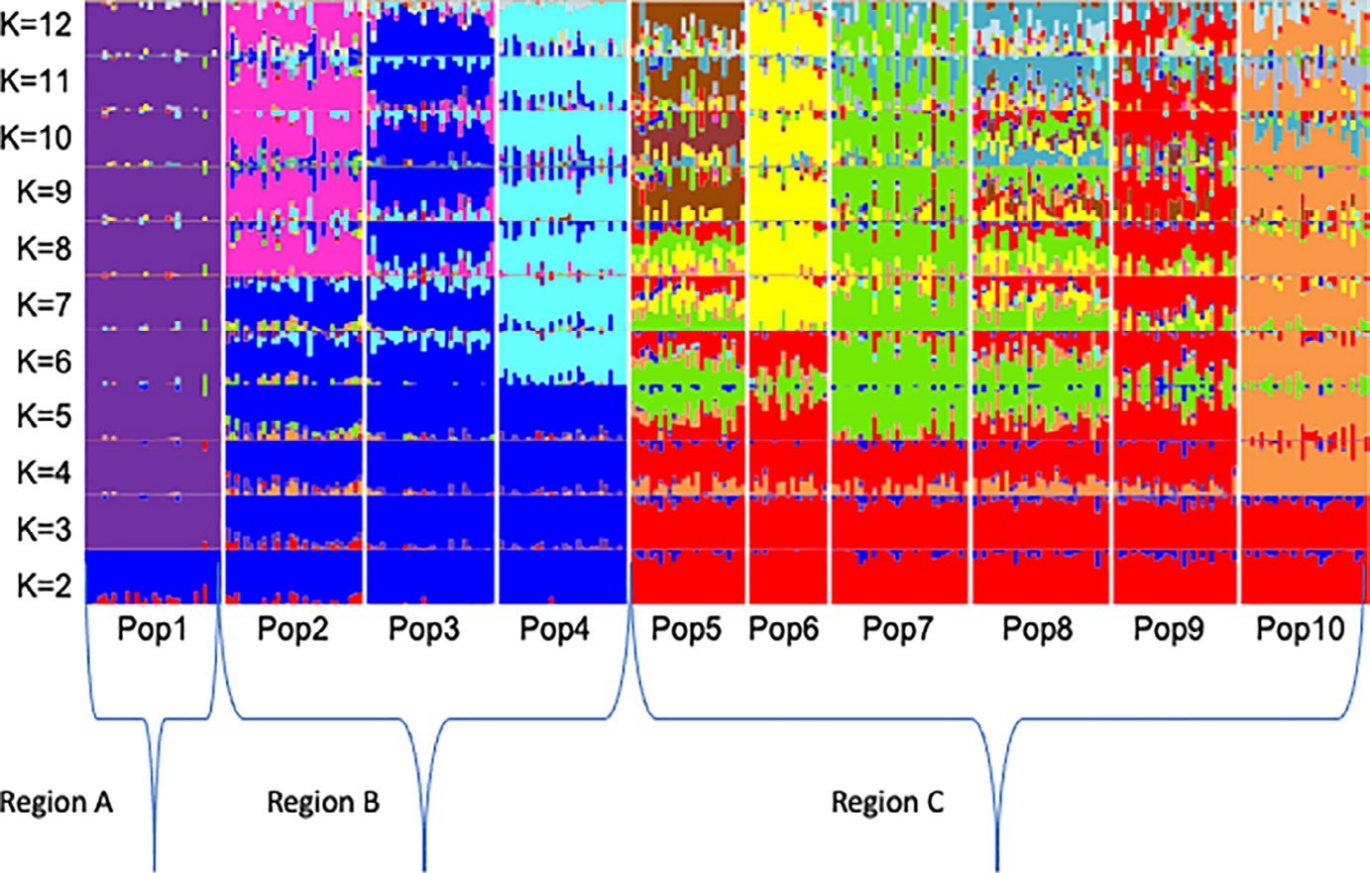
ADMIXTURE analysis for ancestry population clusters (K = 2–12) among the 10 analyzed populations of the marsh fritillary (*Euphydryas aurinia*) (*n* = 273) based on 318 SNPs. Populations are separated by white vertical lines

Populations were strongly clustered into three geographic regions; the minimum CVE value in the ADMIXTURE analysis suggested an optimal number of genotypic clusters for K = 6 (CVE = 0.525) (Figure S3). The graphical visualization of the ADMIXTURE results for the 273 individuals and K ranging from two to 12 clusters is shown in Figure 2. When K = 6, *E. aurinia* is clearly subdivided into different genetic clusters that mainly corresponded with the three geographic regions of the species distribution (i.e., A, B, C) and each of the sampled populations: Region A (population 1) is characterized by a single genetic cluster; Region B is subdivided into two different clusters, one including populations 2 and 3 (dark blue) and an additional cluster exclusively including population 4 (light blue); and Region C genetic subdivision is more complex and characterized by 3 private clusters (red, green and orange clusters). However, while population 10 is mainly characterized by a single genetic component (orange cluster), populations 5 to 9 showed varying levels of genetic admixture of red and green clusters, with populations 5, 7, and 8 showing a predominance of red cluster and 6 and 9 populations a predominance of the green genetic component. Overall, ADMIXTURE results for K ≥ 6 provided similar outputs, but progressively increased the level of genetic resolution within the *E. aurinia* populations, with K = 10 providing a genetic clustering result mainly differentiating each of the sampled marsh fritillary populations with varying levels of genetic admixture (Figure 2).

**FIGURE 3:**
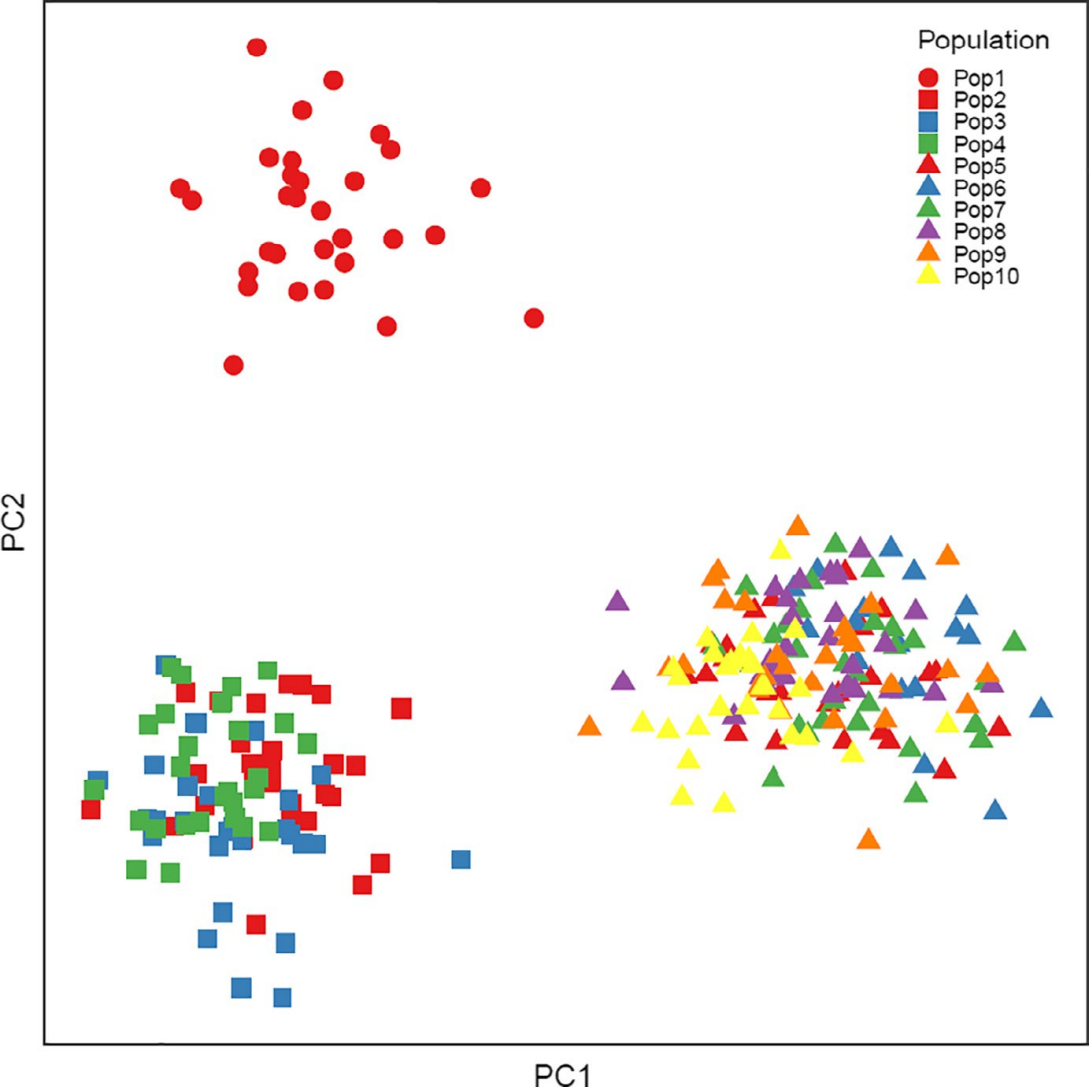
Plot of principal component axes 1 and 2 based on a principal component analysis of the relationship among populations based on genetic distance among 10 populations of the marsh fritillary (*Euphydryas aurinia*). The first PCA axis explained 5.09%, and the second PCA axis explained an additional 3.27% of the variation in the data. Colors and symbols are combined to maximize readability (circles are for Region 1, squares for Region 2, and triangles for Region 3). The sampled populations are listed as in Figure 1: (1) Bruså, (2) Tranum Skydeterræn, (3) Overklitten Sø, (4) Sandmosen, (5) Vågholt Mose, (6) Troldkærvej, (7) Knasborgvej, (8) Videsletengen, (9) Milrimvej, and (10) Strandby

Also, the PCA analysis detected three distinct clusters which were clearly separated by PC1 and PC2 (Figure 3), and they coincide exactly with the three regions A, B, and C of the distribution of the species in Denmark (Figure 1). The genetic relationship among the 10 populations quantified using a principal component analysis (PCA) in which the first two axes (PC1 and PC2) explained 5.09% and 3.27% of the variation, respectively. The first cluster includes only population 1 (region A), the second cluster includes populations 2, 3, and 4 (region B), and the third cluster includes populations 5–10 (region C).

**FIGURE 4:**
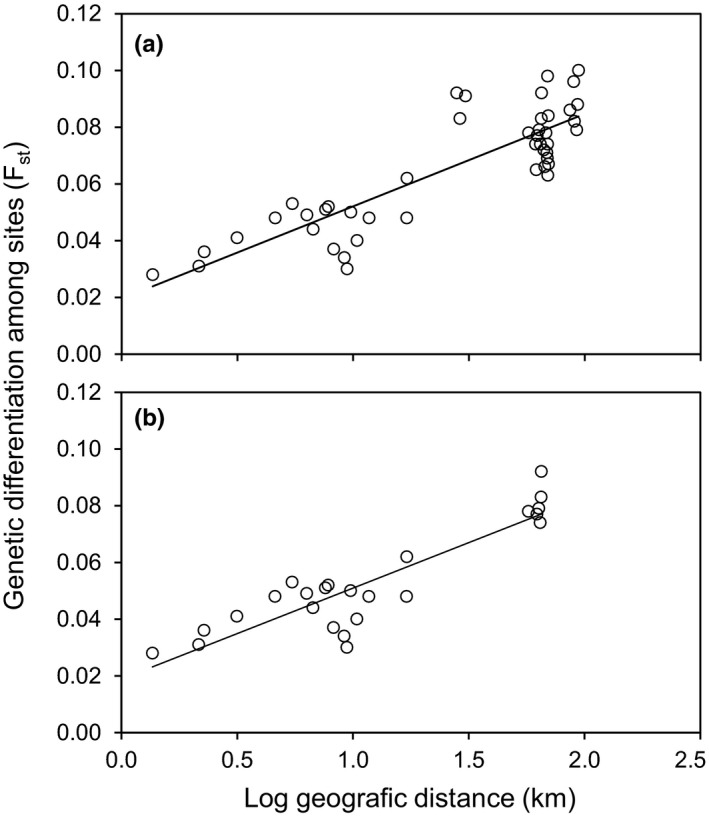
Least square regression of the genetic divergence (*F*_ST_) against log geographic distance in kilometers for (a) all pairwise comparisons among populations (*R* ^2^ = .73, *df* = 43, *p* < .001) and (b) all pairwise comparisons among populations omitting comparisons among the three main regions where the marsh fritillary (*Euphydryas aurinia*) is found in Denmark (*R* ^2^ = .79, *df* = 22, *p* < .001)

Strong evidence for isolation by distance was found across populations; the Mantel tests were found to be highly significant (least square regression analyses; *p* < .001). The regression of genetic divergence (*F*_ST_) against log_10_ of geographic distance in km was highly significant for all pairwise population comparisons (*R* ^2^ = .73, *df* = 43, *p* < .001; Figure 4a) as well as for the subset excluding comparisons among populations in each of the three regions in which the marsh fritillary occurs (*R* ^2^ = .79, *df* = 22, *p* < .001; Figure 4b).

## 4 DISCUSSION

We used a genotyping‐by‐sequencing (GBS) approach to increase the knowledge on the population genetic structure of the critically endangered marsh fritillary butterfly in Denmark. This study documents the identification of an informative SNP loci panel and demonstrates that the GBS approach represents a powerful tool to define genetic relationships at the intraspecific level. Moreover, this SNP panel provides an important genetic resource for further genetic studies of the marsh fritillary and is a cost‐effective and rapid method that can well describe the genetic variability of other nonmodel species with limited genetic resources.

The stringent filtering procedure that we have applied (GQ = 98) (compared with most studies where the GQ filter, is more commonly filtered at 20–40) could have overestimated the heterozygote deficiency and the degree of substructuring of our clusters (Wall et al., 2014). However, the bias introduced is the same for all the clusters and for all the pairwise comparisons between genetic clusters. Hence, our results are still valid for relative comparisons of the genetic parameters among clusters.

The genetic structure of the marsh fritillary in Denmark is clearly affected by limited gene flow among populations. We found significant positive *F*_IS_ values (ranging from 0.1 to 0.228) clearly indicating a high level of inbreeding within populations which indicates substructuring and/or inbreeding due to demographic stochasticity (the stochasticity of the survival of the single larval clusters). The demographic stochasticity is increasing the rate at which inbreeding increased. The consequences of the increased inbreeding are reinforced by the limited dispersal of the butterflies, which is limiting the gene flow among populations. The limited gene flow is also confirmed by the fact that strong evidence for isolation by distance was found, even when accounting for the spatial structure in our data by omitting comparisons among the three regions in which the marsh fritillary is found (Legendre et al., 2015). Detailed capture–mark–recapture studies have demonstrated that most dispersal events are shorter than one kilometer and, only in rare cases, marsh fritillary butterflies disperse more than five kilometers (Johansson et al., 2019; Zimmermann et al., 2011). This suggests that under current levels of fragmentation, isolation by distance can be detected at the same spatial scale as the dispersal capacity of the species. Most of the clusters identified by the ADMIXTURE showed well‐defined genetic clusters coinciding with distinct geographic regions of the distribution of the species in Denmark.

The results of ADMIXTURE show that the populations in regions A and B are more genetically similar while the PCA plot shows a tighter genetic similarity between populations in regions B and C. However, the common approach for detecting the number and subdivision of clusters with the use of ADMIXTURE assumes equilibrium in the genetic conditions, no deviation from HWE, and no linkage disequilibrium within a cluster. Such assumptions are clearly violated in our sample, whereas PCA analyses are not biased by such deviations from genetic equilibrium. Therefore, a cautionary approach should be undertaken when interpreting the genetic relatedness between populations and also the *F*_ST_ distances can mislead as that this genetic‐distance estimator is also based on the assumption of genetic equilibrium within the populations compared. The strong negative relationship found between mean *F*_ST_ and *F*_IS_ provided further evidence for the negative consequences of fragmentation on the genetic variability and the inbreeding level within populations of the marsh fritillary in Denmark.

Our results suggest that further efforts are needed to maintain genetic diversity in this species in Denmark. Source‐sink dynamics will effectively increase mortality from isolated populations because dispersing individuals will be unable to locate suitable habitat. In addition, reduced mixing of populations will affect genetic diversity and ultimately cause inbreeding (Pertoldi et al., 2007; Sigaard et al., 2008). A breeding program for the marsh fritillary in the United Kingdom demonstrated a strong positive effect on reproduction by mixing populations from Cumbria and Scotland into a hybrid stock (Porter & Ellis, 2011). Since all populations in our study from Denmark were inbred, similar positive effects of mixing populations in Denmark could be expected. Although several strongholds for the marsh fritillary exist in Denmark, establishing a breeding program could be relevant. Hybrid populations could serve as a way to secure genetic diversity and may be relevant for potentially translocating the species to unoccupied regions of Denmark with suitable habitat networks (Brunbjerg et al., 2017).

Population structure has been studied in many butterfly species using microsatellite markers (Saccheri et al., 2004; Smee et al., 2013; Vandewoestijne et al., 2011; Zeisset et al., 2005). However, due to the formidable challenges involved in developing informative microsatellite markers (Nève & Meglécz, 2000), most studies have relied on a small number of markers with limited resolution (Sigaard et al., 2008). Methods like GBS can help guide priorities for other species in similar situations (Elshire et al., 2011).

We found isolation by distance for patches across a wide range of distances among populations. We expected that populations in the three regions investigated to be completely isolated from each other, but the correlation between geographic distance and genetic divergence suggests that some gene flow may occur among these regions. The genetic differentiation observed among the three regions is, however, compatible with the expectation in species with poor dispersal capacity. Given the fact that the populations are separated by unsuitable habitats, we expect that only very rare dispersal events from one region to another is occurring. Long and relatively rare dispersal events have been detected among populations of marsh fritillary in the Czech Republic populations (Zimmermann et al., 2011). Further studies are needed to elucidate how variation in habitat characteristics like resource availability in different life stages affect propensity for dispersal and to demonstrate that such long‐distance dispersal among regions does occur. In order to mitigate further losses of genetic diversity, conservation efforts targeting rare species like the marsh fritillary existing in fragmented landscapes like in Denmark should concentrate on further enhancing connectivity among existing habitat patches.

## CONFLICT OF INTEREST

The authors have no conflict of interest.

## AUTHOR CONTRIBUTIONS

**Cino Pertoldi:** Conceptualization (lead); Data curation (equal); Formal analysis (equal); Funding acquisition (equal); Investigation (equal); Methodology (lead); Project administration (equal); Resources (equal); Software (equal); Supervision (equal); Validation (lead); Visualization (equal); Writing‐original draft (lead); Writing‐review & editing (equal). **Aritz Ruiz‐Gonzalez:** Conceptualization (equal); Data curation (equal); Formal analysis (equal); Funding acquisition (equal); Investigation (equal); Methodology (equal); Project administration (equal); Resources (equal); Software (equal); Supervision (equal); Validation (equal); Visualization (equal); Writing‐original draft (equal); Writing‐review & editing (equal). **Simon Bahrndorff:** Conceptualization (equal); Data curation (equal); Formal analysis (equal); Funding acquisition (equal); Investigation (equal); Methodology (equal); Project administration (equal); Resources (equal); Software (equal); Supervision (equal); Validation (equal); Visualization (equal); Writing‐original draft (equal); Writing‐review & editing (equal). **Nanna Renee Lauridsen:** Conceptualization (supporting); Data curation (supporting); Formal analysis (supporting). **Thøger Nisbeth Henriksen:** Conceptualization (supporting); Data curation (supporting). **Anne Eskildsen:** Conceptualization (equal); Data curation (equal); Formal analysis (equal); Funding acquisition (equal); Investigation (equal); Methodology (equal); Project administration (equal); Resources (equal); Software (equal); Supervision (equal); Validation (equal); Visualization (equal); Writing‐original draft (equal); Writing‐review & editing (equal). **Toke Thomas Høye:** Conceptualization (equal); Data curation (equal); Formal analysis (equal); Funding acquisition (equal); Investigation (equal); Methodology (equal); Project administration (equal); Resources (equal); Software (equal); Supervision (equal); Validation (equal); Visualization (equal); Writing‐original draft (equal); Writing‐review & editing (equal).

## Supporting information

Appendix S1

## Data Availability

Data for this study are available at Dryad: https://datadryad.org/stash/share/XetcrVN2qTVd3aRg1eK4LxAmZXKBegZtm0skGawIr1o.
