# Supplementary material for: Strong isolation by distance among local populations of an endangered butterfly species (Euphydryas aurinia)
Source: Ecol Evol. 2021 Aug 13;11(18):12790–800. doi: 10.1002/ece3.8027 (PMC8462152; doi:10.1002/ece3.8027)
Supplement: Supplementary file 1 — Appendix S1 [file ECE3-11-12790-s001.docx]

**Electronic appendix**

**Appendix Figure 1.** Fragment size distribution of GBS libraries using three restriction enzymes a) ApeKI; b) EcoT22I and c) PstI). The x-axis represents elution time and the y-axis shows ﬂuorescence units. Numbers below hatch marks on the x-axis indicate fragment size (bp). Tall peaks at 15 and 1500 bp are size standards.

a)


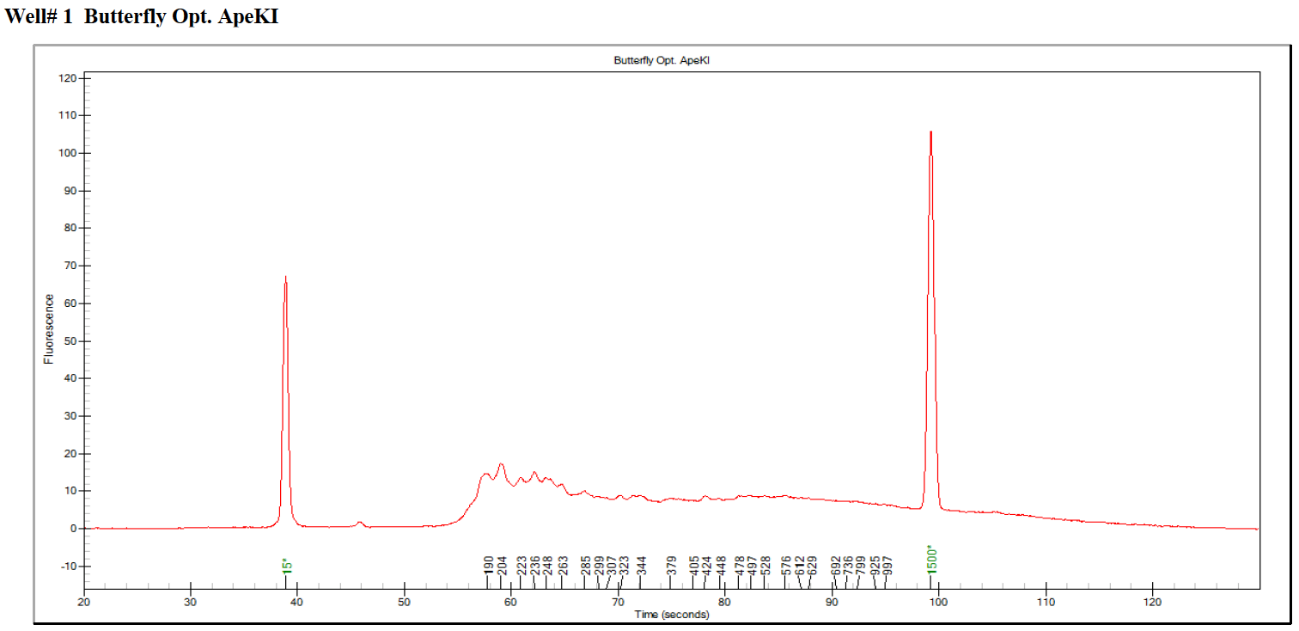


b)


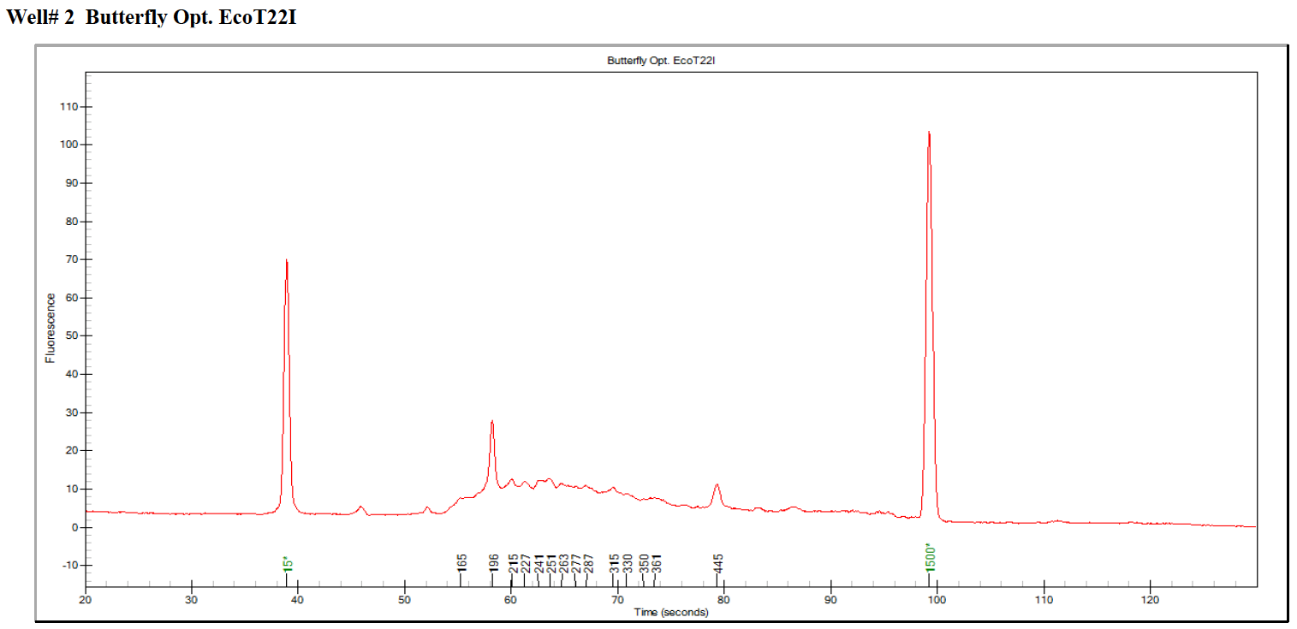


c)
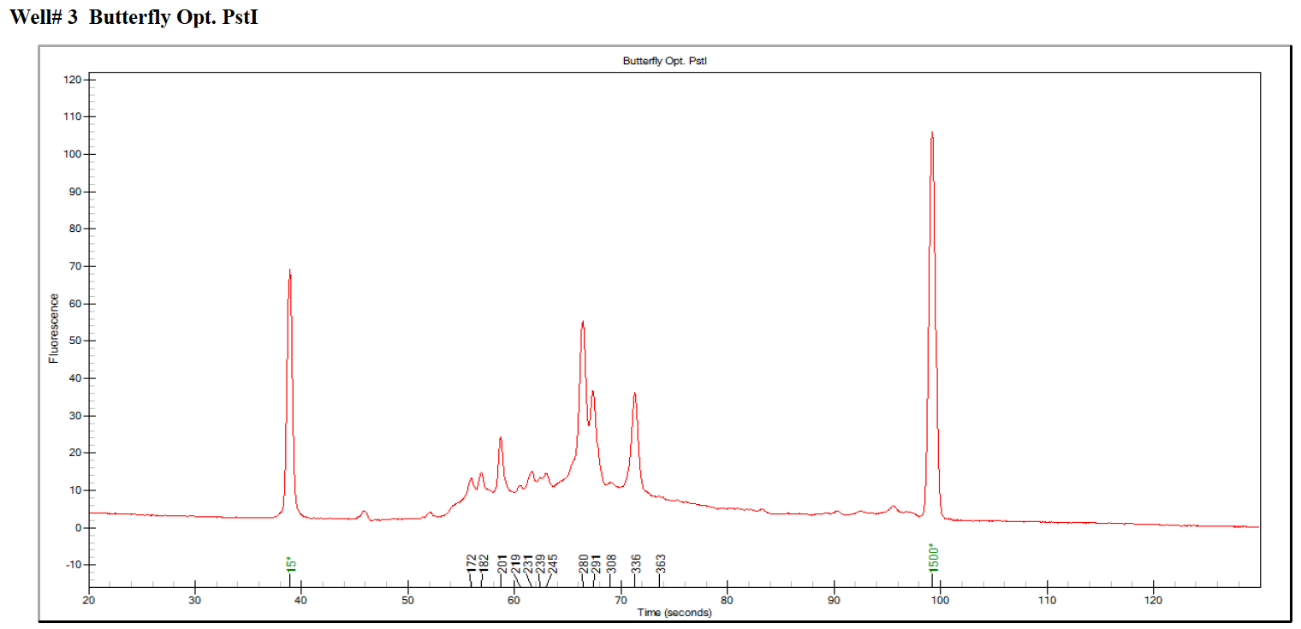


**Appendix Figure 2**. Post assembling filtering.

The resulting raw SNPs dataset from the UNEAK pipeline (30,137 bi-allelic SNP loci, n=280 individuals) were further filtered using Golden Helix SNP and Variation Suite (SVS Version 7.2.2, Golden Helix, Bozeman, MT) and PLINK v1.07 (Purcell et al., 2007) softwares.

First, the dataset was filtered by the application of genotype-level filters to remove genotypes with low read depths (RD) and/or low genotype quality (GQ) reducing the panel to 832 snps.

Thus, genotypes with RD ≤ 4x and GQ ≤ 98 were considered as missing. Later, we removed all SNPs and individuals with call-rates < 80%. (only retained 273 individuals out of 280; and obtained 539 snps.

In addition, SNPs with a minor allele frequency (MAF) < 0.05 were removed (SNPs=342).

Loci with a mean observed heterozygosity greater than 0.6 were also discarded to filter out potential paralogs (snps = 325).

The SNP set was also pruned for linkage disequilibrium (LD) by excluding markers in strong LD (pairwise genotype correlation r^2^ > 0.5) in a window of 50 SNPs (sliding window overlap 10 SNPs at a time) (snps=318).

After the complete filtering procedure 318 SNPs were maintained in the matrix for 273 individuals with an overall call rate of 93.57%.

**Appendix Figure 3**

Cross-validation error (CVE) test, performed 100 times for each potential number of ancestry clusters K in the range 1–12, in which the best-fitting K was selected based on minimizing CVE. The lowest CVE value (0.525) was found for K = 6 and is indicated by the horizontal hatched line.
